# Low-Temperature Fabrication of Refractory Thin Films via Electric Field and Contact Stress-Activated Sintering of Nanoparticles: An *In Situ* Study

**DOI:** 10.1021/acs.nanolett.5c04675

**Published:** 2025-11-17

**Authors:** Bunty Tomar, Pranjal Nautiyal

**Affiliations:** School of Mechanical and Aerospace Engineering, 7618 Oklahoma State University, Stillwater, Oklahoma 74078, United States

**Keywords:** Nanostructured thin film, Sintering, Tribosintering, Electric field, WC-Co nanocomposite, Nanomanufacturing

## Abstract

Refractory nanocomposite films are employed to prevent wear of mechanical components under harsh conditions; however, they are traditionally manufactured at elevated temperatures, causing undesirable microstructural transformations. This study presents a novel electric field- and contact stress-activated sintering process for fabricating tungsten carbide (WC-Co) films at low temperatures (100 °C). In this process, nanoparticle-containing “inks” are supplied to stressed sliding/rolling interfaces, while simultaneously applying direct current. Elevated stresses (>1 GPa) and electric currents (2–5 A) drive nanoparticle sintering on contacting surfaces, generating thin films within minutes. Here, we present the results of an *in situ* study of the kinetics of sintering using optical interferometry. The application of electric currents enhanced film thickness, reduced surface roughness, and increased the fraction of WC incorporated into the film. Co played a critical role in film nucleation by forming a deformable matrix for trapping hard WC nanoparticles. This manufacturing approach provides a rapid, low-temperature pathway for fabricating nanostructured films.

## Graphical abstract

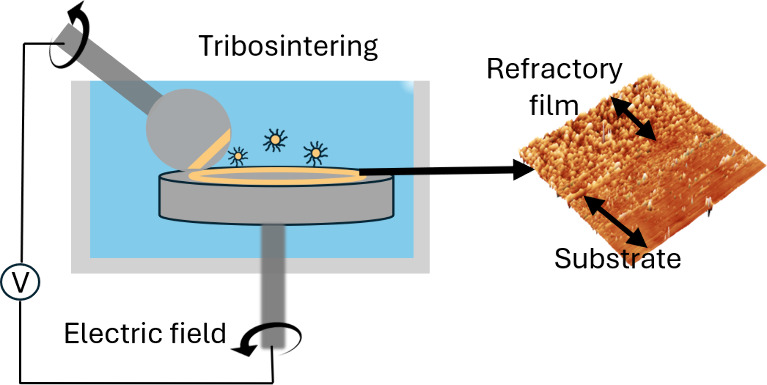

Refractory films and coatings, such as tungsten carbide–cobalt (WC-Co)^1,2^ nanocomposites, are desirable for surface engineering of mechanical components due to their superior hardness, wear resistance, and thermal and microstructural stability.^3,4^ However, fabrication of these films requires high temperature processes, such as thermal spray (e.g., plasma spray, flame spray, and high-velocity oxygen fuel spray^5,6^ ) and powder metallurgy (e.g., conventional sintering,^7,8^ pulsed plasma sintering,^9^ and spark plasma sintering^10^), which involve processing temperatures exceeding 1000 °C. These high-temperature processes generate pores and microcracks in the microstructure^11^ and can undermine the mechanical properties.^12,13^ Moreover, it has been reported that elevated temperatures during manufacturing lead to the decarburization of WC and loss of dissolved C as CO/CO_2_. This reduces the overall WC content in the coating and leads to the entrapment of some dissolved C within the Co binder. This leads to a reduction in the hardness and wear resistance of the coatings.^14^ Additionally, high processing temperatures can lead to the formation of heat-affected zones, promoting grain growth, phase transformations, and secondary phase precipitation, all of which can compromise the mechanical properties of the films.^15–17^

Recently, tribosintering has emerged as an effective process for fabricating thin films at low temperatures. In this process, mechanical stresses at rolling/sliding contacts drive the consolidation of nanoparticles into dense, nanocrystalline films.^18^ The mean compressive and shear stresses generated at sliding contacts are on the order of hundreds of MPa and can also exceed a GPa, which activate the sintering of nanoparticles on the contacting surfaces.^19,20^ Several studies have reported tribosintering of metal oxide thin films, including ZrO_2_,^20–25^ TiO_2_,^26^ and ZnO.^27^ However, the mechanical properties of tribosintered films do not match up to their bulk counterparts.^20,28^ These films also suffer from poor adhesion, rapid wear, and delamination when exposed to extreme stresses at sliding contacts.^29^

One possible strategy to overcome these challenges is the application of electric fields during tribosintering. We hypothesize that electric fields at sliding–rolling contacts can accelerate the sintering of nanoparticles, producing denser and stronger thin films. Similar effects have been observed in spark plasma sintering (SPS),^30^ where electric current induces pore elimination and rapid densification due to enhanced atomic diffusion through localized Joule heating and electromigration.^31–33^ The role of electric fields in promoting the tribosintering of nanoparticles remains unexplored.

In this work, we report electric field-assisted tribosintering of WC-Co thin films on steel substrates. In this process, a nanoparticle-containing ink (nanofluid) is supplied to sliding/rolling interfaces, where a combination of highly concentrated mechanical stresses and electric fields activates the sintering of nanoparticles under low temperatures. We show that applying a modest electric current across the contact not only promotes WC-Co film growth rate and thickness but also alters the film composition and surface roughness. This study reveals a fundamentally new route to engineer nanostructured refractory films at low processing temperatures (100 °C).

A ball-on-disc tribometer capable of generating electrified sliding/rolling contact^34^ was used as the testbed to investigate electric field-assisted tribosintering of WC-Co nanoparticles. An oil-based ink containing dispersed WC-Co nanoparticles was supplied to the sliding–rolling interface (illustrated in Figure 1a). The applied mechanical stresses (1.12 GPa maximum Hertzian contact pressure) and direct currents (2 and 5 A) activated the sintering of the nanoparticle films. This testbed is equipped with a Spacer Layer Imaging Method (SLIM) system^35^ (Figure 1b), which captured interference images at regular intervals (Figure 1c), enabling *in situ* measurement of film growth kinetics. A detailed description of methods and experimental parameters is provided in the Supporting Information.

**Figure 1:**
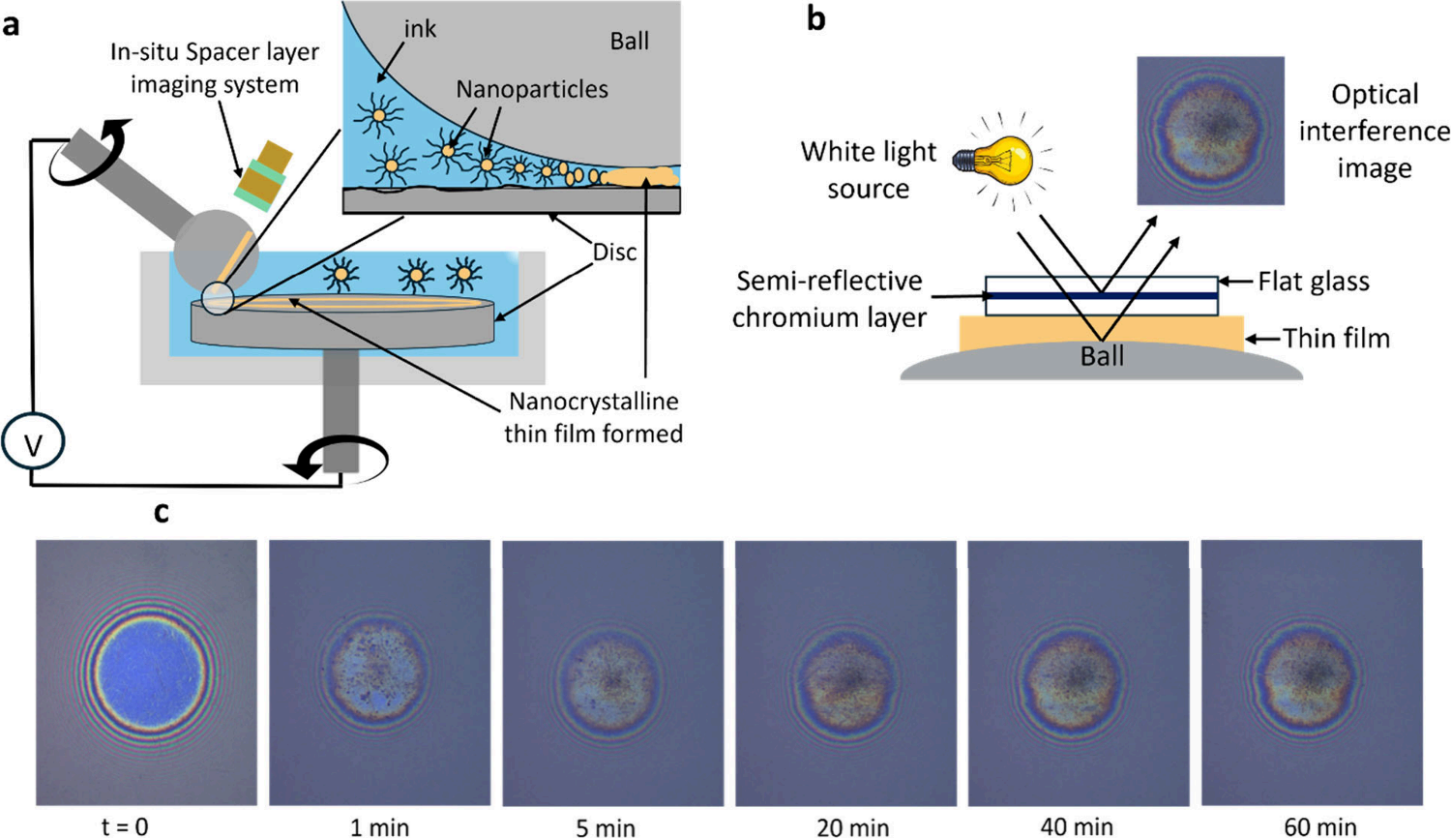
Testbed for *in situ* investigation of electric field-assisted tribosintering of WC-Co films. **a,** Schematic of the ball-on-disc tribometer testbed used for studying field-assisted tribosintering. The WC-Co nanoparticles are supplied to the ball/disc interface by using a carrier fluid (ink). An electric circuit supplies the current to the ball/disc interface. **b,** Schematic representation of white-light-based optical interference imaging used to measure thin film evolution on the ball specimen. **c,** *In* *situ* optical interferometry images showing nucleation and growth of a WC-Co film on the ball specimen for the 0 A condition. Images for 2 and 5 A conditions are shown in Figure S1.

At 0 A, the contact stress-activated sintering resulted in the formation of a thin film that rapidly developed within the first 10 min of sliding/rolling contact cycles (Figure 2a). Beyond this point, the film thickness stabilized, reaching an average value of 13.5 ± 0.7 nm (Figure 2b). This growth behavior is consistent with previous tribosintering studies, which also report a self-limiting growth of thin films.^19–21,36^ In contrast, under the 2 A condition, film growth exhibited two distinct stages: a rapid growth phase was observed during the initial 10 min, followed by a slower growth regime extending up to 30 min (Figure 2a). The final average film thickness was 17.8 ± 0.7 nm (Figure 2b), confirming that the introduction of electric current enhances tribosintering. Under the 5 A condition, a more pronounced electric field effect was observed. The film thickness exhibited a steady increase for up to 20 min (Figure 2a) before reaching a plateau at 24.8 ± 1.0 nm (Figure 2b). The film thickness evolution in the initial growth regime fitted well to a power-law function (see the zoomed-in plot in Figure 2a). The instantaneous film growth rates were determined by differentiating the fitted curves with respect to time (see eqs S5 and S6 in the Supporting Information) and then averaged to obtain the mean film growth rate (Figure 2c). The film growth rate increased with the applied direct current, suggesting a field-activated sintering mechanism.

**Figure 2:**
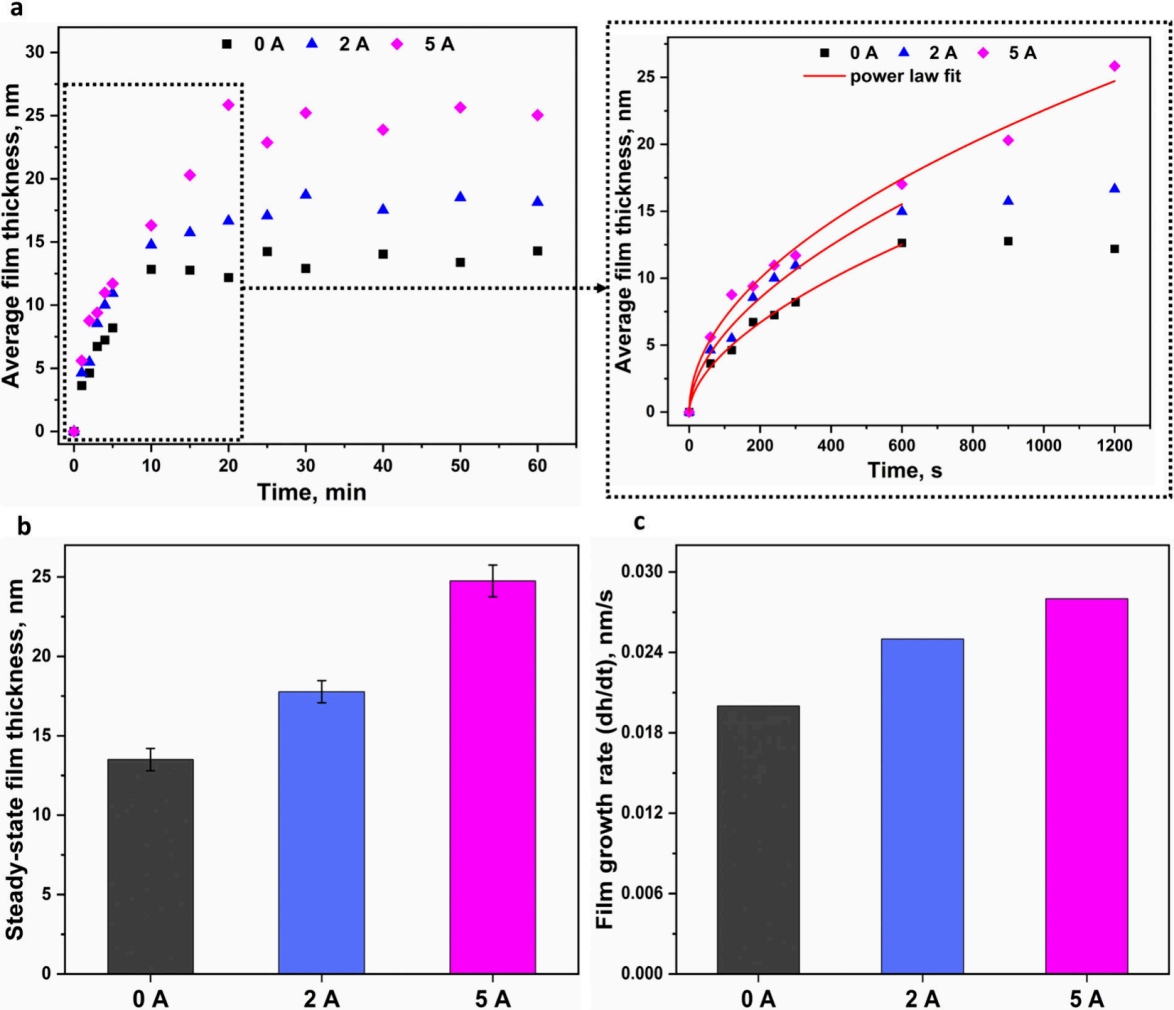
Growth evolution behavior of WC-Co thin films. **a,** Representative thin film thickness evolution as a function of time for 0, 2, and 5 A test conditions, highlighting the influence of electric current on film growth kinetics. Zoomed-in plot shows film thickness evolution versus time during the initial growth phase with power law fits. **b,** Comparison of steady state film thickness demonstrating that increasing the electric field strength enhances the final film thickness. The error bars represent standard deviation of film thickness after saturation. **c,** Average film growth rates for all test conditions calculated from the derivatives of the power law fits.

Other field-assisted sintering methods, such as SPS, also report that electric fields can promote sintering by inducing localized heating, enhanced atomic mobility, and faster neck formation between particles.^32,37^ Despite these similarities, there are fundamental differences between the field-assisted tribosintering in this work and SPS. Unlike SPS, which typically requires processing temperatures exceeding 1000 °C^10^ and elevated electric fields (∼1000 A^38^), the tribosintering approach in this work enabled film formation at just 100 °C and under modest electric currents (up to 5 A). These differences could be attributed to the absence of shear stresses and relatively lower compressive stresses (<100 MPa) in SPS.^38^ In contrast, the electric field-assisted tribosintering method in this work involved a combination of elevated compressive (>1 GPa) and shear stresses (>100 MPa) at sliding/rolling interfaces. A combination of highly concentrated stresses and electric currents activated the consolidation of nanoparticle films under modest temperatures in this work.

To further elucidate the role of the electric field in promoting film densification, *ex* *situ* atomic force microscopy (AFM) was conducted on the steel discs to capture nanoscale topography of the thin films formed under 0, 2, and 5 A applied currents. Across all conditions, AFM imaging revealed the formation of a uniform film on the running track, as shown in the large-area scans (100 μm × 100 μm, top row) in Figure 3. The zoomed-in images (5 μm × 5 μm, bottom row in Figure 3) revealed small scale features of WC-Co films. This morphology arises from the nucleation and growth of nanoscale clusters^20,21^ due to contact stresses and frictional heating at multiasperity sliding/rolling contacts. Such morphology has also been reported for other nanoceramic films fabricated by tribosintering.^19^ The films exhibited an RMS roughness of 16.5, 17.0, and 10.0 nm at 0, 2, and 5 A, respectively. The 5 A condition produced the smoothest film. We attribute this to electric current-induced plastic deformation of surface asperities, a mechanism known as electroplasticity.^39,40^

**Figure 3:**
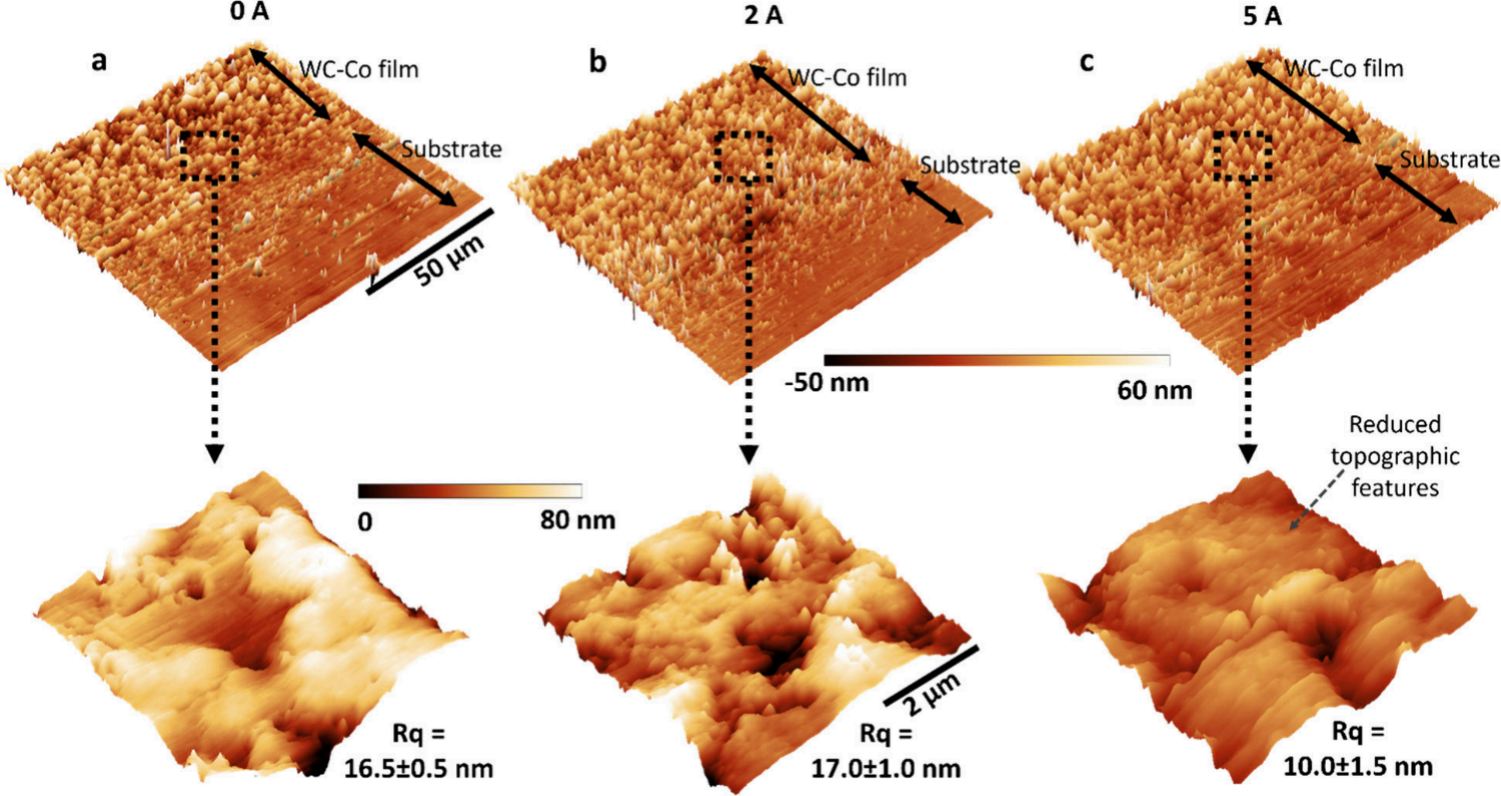
AFM characterization of WC-Co thin films manufactured under varying electric field conditions. AFM images of WC-Co films generated at **a,** 0 A, **b,** 2 A, and **c,** 5 A test conditions.

To gain insight into the microstructure and composition of thin films, scanning electron microscopy (SEM) and energy-dispersive spectroscopy (EDS) were conducted (Figure 4). These analyses were aimed at elucidating the underlying mechanisms of electric field-assisted tribosintering. Despite differences in film thickness across the 0, 2, and 5 A test conditions driven by electric current, the width of the WC-Co films remained nearly constant at ∼340 μm for all conditions (Figure 4a). This observation indicates that film width is not significantly influenced by the electric field but is rather governed by the Hertzian contact area, which is a function of the applied normal load and contact geometry (see Supporting Information S1.1).

**Figure 4:**
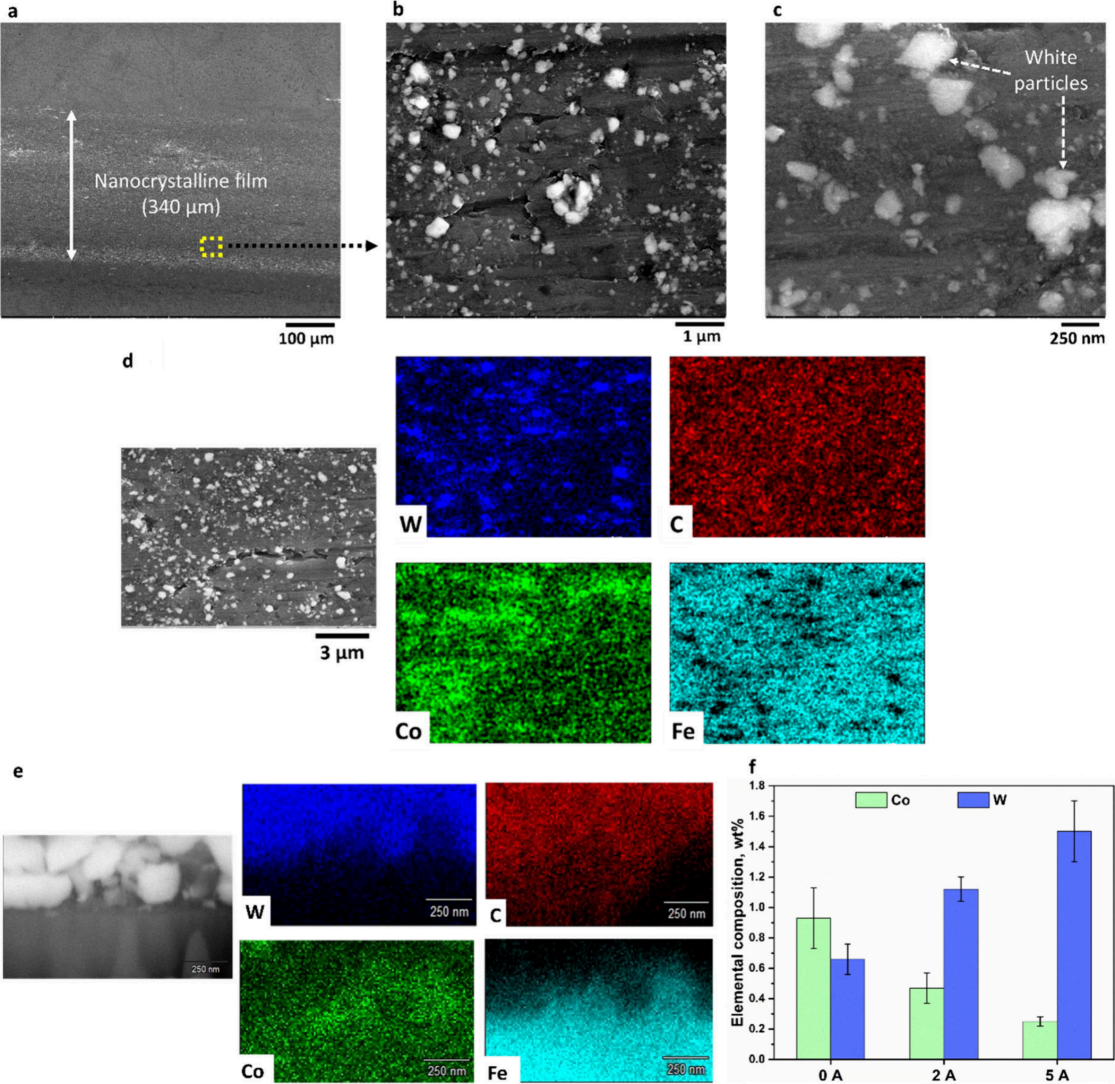
SEM and EDS analyses of WC-Co thin films sintered on a steel substrate. **a,** Top view SEM image a WC-Co film formed on the steel substrate at 5 A. The lateral film width (∼340 μm) remained consistent across all test conditions. **b, c,** Higher magnification SEM images reveal the presence of distinct white phases embedded within a darker continuous matrix. **d,** EDS elemental mapping of a film formed at 5 A identifies the shiny white features as WC particles and the dark matrix as Co and Fe. **e,** Cross-section SEM image and EDS maps showing the structure and composition of a WC-Co film formed at 5 A. The SEM image shows an intimate interface between the film and the substrate. **f,** Histogram showing elemental composition of W and Co in thin films sintered at 0, 2, and 5 A test conditions. An increase in WC content and a corresponding decrease in Co content are observed with increasing electric field.

The films were composed of WC and Co phases, which was confirmed by EDS mapping (Figure S2). At higher magnification, SEM micrographs revealed a dual-phase microstructure, consisting of white nanophases embedded in a darker continuous matrix (Figure 4b). These distinct contrasts are indicative of compositional heterogeneity. The size of the white particles was up to ∼300 nm, as shown in Figure 4c. Elemental mapping via EDS confirmed the presence of Fe, W, Co, Cr, and C as the primary constituents (Figure 4d). Among these, Fe was most abundant and was detected from the substrate underneath the thin film, due to the larger interaction volume of the electron beam^41^ than the film thickness. Importantly, Co and WC exhibited clear phase separation, with a darker region identified as Co, forming the continuous phase, and white phases confirmed as WC appearing as clusters of nanoparticles embedded in the Co matrix (see Figure S3).

The internal structure of the film and the interface between the film and the substrate were revealed by sectioning the film using focused ion beam (FIB) milling (Figure 4e). The cross-sectional SEM imaging showed intimate bonding between the film and the substrate. EDS mapping revealed intermixing of Fe, WC, and Co across the interface region, confirming the nanoparticles were implanted into the steel substrate. Moreover, the cross-sectional imaging shows the film is dense, without significant porosity, which is desirable for mechanical properties.

To elucidate the role of the Co phase in tribosintering of nanocomposite films, we also investigated a film formed by only WC nanoparticles under identical electric field and contact conditions. Unlike WC-Co, WC films were sparse, with an average thickness of less than 5 nm at 5 A (Figure S4). The contact track exhibited prominent scars exceeding 100 μm in length and over 15 μm in width (Figure S5), indicating severe abrasive wear. In the absence of a soft, ductile binder phase like Co, the hard WC nanoparticles likely acted as abrasive agents under high contact stress (∼1.12 GPa), leading to substrate plowing and material removal. This striking difference highlights that the presence of a soft, ductile phase like Co is essential for tribosintering of refractory nanocomposites. Under applied mechanical stresses, Co undergoes plastic deformation, spreading across the contact interface and forming a compatible interfacial layer. This soft layer serves as a mechanically accommodating matrix for refractory WC nanoparticles to physically embed, forming a WC-Co nanocomposite film. In contrast, the WC-only system lacks this ductile phase. The brittle WC particles do not plastically deform under stress, and without a ductile phase to facilitate embedding, the particles fail to form a dense film (Figure S6). As a result, WC nanoparticles (without the Co phase) tend to form only isolated clusters on the steel surface. This is consistent with previous reports on SPS, where ductile binder phases promote densification, mechanical integrity, and phase homogeneity.^42^

To understand the sintering mechanism of WC-Co, we quantified the relative fractions of WC and Co phases in the microstructure of thin films generated under different applied electric currents. As current increased from 0 to 5 A, Co content in the films decreased, while WC content increased (Figure 4f). This compositional shift is further corroborated by the increasing abundance of white, WC-rich particles visible in the SEM micrographs (see Figure S7). The application of electric current during tribosintering likely initiated the formation of thermal hotspots across the contact.^43^ These hotspots arise from field-induced Joule heating at the particle–particle interface where resistive heating is amplified, creating regions of elevated temperature that far exceed the average temperature. This localized overheating promotes the sintering of WC nanoparticles at sliding contacts. Therefore, higher currents promote incorporation of WC into the thin film. Concurrently, the softer Co phase will be removed due to abrasive wear as the content of the WC particles in the film increases. These findings underscore the importance of electric fields in designing the microstructure of nanocomposite films generated via tribosintering. Increasing the incorporation of the hard WC phase in the film is desirable for enhanced hardness and wear resistance.^44^ Our future studies will focus on correlating the mechanical properties of WC-Co films with electric field-activated sintering mechanisms.

In summary, we presented electric field- and contact stress-activated tribosintering as a novel method for fabricating refractory nanocomposites at low temperatures. This method exploits concentrated stresses and electric currents at sliding and rolling interfaces to sinter nanoparticle thin films on contacting metal surfaces. Our results reveal that electric currents play a crucial role in sintering kinetics and thin film microstructure. First, they promote vertical film growth. Second, they reduce surface roughness by promoting the plastic deformation of surface asperities. Third, they increase the incorporation of WC particles into thin films. The presence of a ductile Co phase was found to be essential for dense film formation, as it provides a deformable matrix for embedding hard WC particles, a phenomenon absent when only WC nanoparticles were used. Altogether, these synergistic effects enable the formation of thicker and smoother nanocrystalline films at contact interfaces. The clear correlation between applied electric current and film growth kinetics underscores the potential of field-assisted tribosintering as a scalable route for fabricating refractory thin films at low temperatures. This method can be scaled to coat large areas by using larger diameter probes or by using alternative contact geometries, such as a cylinder-on-flat, which generate line contacts (see Figure S8), significantly enhancing the contact area for sintering thin films. Future work will explore the effects of nanoparticle size and composition,^7,45^ the relative compositions of ceramic and binder phases, and other classes of technologically important refractory materials, such as high-entropy ceramics and composites.^46–48^ This rapid and efficient nanomanufacturing approach could be employed for surface engineering in extreme applications, such as hypersonics, geothermal and wind energy, and spacecraft, where nanostructured refractory films can protect mechanical components from wear and failure under extreme temperatures, elevated stresses, and corrosive environments.

## Supplementary Material



## Notes

Methods (*in situ* film growth experiments and thin film characterization), materials, *in situ* optical interferometry images, EDS maps of WC-Co films, EDS point results of WC-Co films, WC-Co and WC film thickness comparison, SEM images of WC-only and WC-Co films, schematic of strategies for coating large areas (PDF)

## References

[1] Zhu Y. C., Ding C. X., Yukimura K., Xiao T. D., Strutt P. R. (2001). Deposition
and Characterization of Nanostructured WC–Co Coating. Ceram. Int..

[2] Qiao Y., Liu Y., Fischer T. E. (2001). Sliding and Abrasive
Wear Resistance of Thermal-Sprayed
WC-CO Coatings. J. Therm. Spray Technol..

[3] Yu L., Khor K., Li H., Pay K., Yip T., Cheang P. (2004). Restoring WC in Plasma
Sprayed WC–Co Coatings
through Spark Plasma Sintering (SPS). Surf.
Coat. Technol..

[4] Wood R. J. K. (2010). Tribology
of Thermal Sprayed WC–Co Coatings. Int.
J. Refract. Met. Hard Mater..

[5] Garfias
Bulnes A., Albaladejo Fuentes V., Garcia Cano I., Dosta S. (2020). Understanding the Influence of High Velocity Thermal Spray Techniques
on the Properties of Different Anti-Wear WC-Based Coatings. Coatings.

[6] Matthews S., Ansbro J., Berndt C. C., Ang A. S. M. (2021). Carbide
Dissolution
in WC-17Co Thermal Spray Coatings: Part 1-Project Concept and as-Sprayed
Coatings. J. Alloys Compd..

[7] Barja A. M., Ferrari B., Tejado E., Pastor J. Y., Sanchez-Herencia A. J. (2025). Enhanced
Densification and Microstructural Refinement in Low-Ni WC Composites:
Conventional Sintering Optimisation. J. Alloys
Compd..

[8] Raihanuzzaman R. M., Han S.-T., Ghomashchi R., Kim H.-S., Hong S.-J. (2015). Conventional
Sintering of WC with Nano-Sized Co Binder: Characterization and Mechanical
Behavior. Int. J. Refract. Met. Hard Mater..

[9] Rosinski M., Michalski A. (2012). WCCo/CBN Composites
Produced by Pulse Plasma Sintering
Method. J. Mater. Sci..

[10] Liu K., Wang Z., Yin Z., Cao L., Yuan J. (2018). Effect of
Co Content on Microstructure and Mechanical Properties of Ultrafine
Grained WC-Co Cemented Carbide Sintered by Spark Plasma Sintering. Ceram. Int..

[11] Özorak C., Islak S. (2024). Microstructure, Wear
and Corrosion Properties of Cu–SiC/WCCo
Composite Coatings on the Cu Substrate Surface by Plasma Spray Method. Mater. Chem. Phys..

[12] Qiao X., Wang Y. M., Weng W. X., Liu B. L., Li Q. (2018). Influence
of Pores on Mechanical Properties of Plasma Sprayed Coatings: Case
Study of YSZ Thermal Barrier Coatings. Ceram.
Int..

[13] Zhang H., Xie Y., Huang L., Huang S., Zheng X., Chen G. (2014). Effect of
Feedstock Particle Sizes on Wear Resistance of Plasma Sprayed Fe-Based
Amorphous Coatings. Surf. Coat. Technol..

[14] Schwetzke R., Kreye H. (1999). Microstructure and
Properties of Tungsten Carbide Coatings Sprayed
with Various High-Velocity Oxygen Fuel Spray Systems. J. Therm. Spray Technol..

[15] Zhu B., Hu Q., Zeng X., Meng L., Liu X., Xu G. (2023). Effect of
Intrinsic Thermal Cycles on the Microstructure and Mechanical Properties
of Heat Affect Zones in Laser Cladding Coatings on Full-Scale Rails. Surf. Coat. Technol..

[16] Araujo P., Chicot D., Staia M., Lesage J. (2005). Residual Stresses and
Adhesion of Thermal Spray Coatings. Surf. Eng..

[17] Jiang H. G., Lau M. L., Lavernia E. J. (1998). Grain Growth Behavior of Nanocrystalline
Inconel 718 and Ni Powders and Coatings. Nanostructured
Mater..

[18] Kato H., Komai K. (2007). Tribofilm Formation and Mild Wear
by Tribo-Sintering of Nanometer-Sized
Oxide Particles on Rubbing Steel Surfaces. Wear.

[19] Thrush S. J., Comfort A. S., Dusenbury J. S., Han X., Wang X., Qu H., Barber G. C. (2021). Study of Pressure
Dependence on Sinterable Zirconia
Nanoparticle Tribofilm Growth. Tribol. Int..

[20] Khare H. S., Lahouij I., Jackson A., Feng G., Chen Z., Cooper G. D., Carpick R. W. (2018). Nanoscale
Generation of Robust Solid
Films from Liquid-Dispersed Nanoparticles via in Situ Atomic Force
Microscopy: Growth Kinetics and Nanomechanical Properties. ACS Appl. Mater. Interfaces.

[21] Thrush S. J., Comfort A. S., Dusenbury J. S., Nautiyal P., Elinski M. B., Carpick R. W., Demas N. G., Gould B. J., Han X., Wang X., Qu H., Barber G. C. (2022). Growth and Morphology
of Thermally Assisted Sinterable Zirconia Nanoparticle Tribofilm. Tribol. Int..

[22] Khare H. S., Gosvami N. N., Lahouij I., Milne Z. B., McClimon J. B., Carpick R. W. (2018). Nanotribological
Printing: A Nanoscale Additive Manufacturing
Method. Nano Lett..

[23] Lahouij I., Gould B., Demas N., Greco A., Chen Z., Cooper G. D., Jackson A., Carpick R. W. (2022). Inhibition of Micro-Pitting
by Tribofilm-Forming ZrO2 Nanocrystal Lubricant Additives: A Micro-Pitting
Rig and Transmission Electron Microscope Study. Tribol. Lett..

[24] LaMascus P., Elinski M. B., Delghandi D., Nautiyal P., Griffin J., Zheng L., Jackson A., Wiacek R. J., Carpick R. W. (2024). Competition
Between Growth and Removal in Zirconia Nanocrystal-Derived Tribofilms:
The Role of Co-Additives. Tribol. Lett..

[25] Elinski M. B., LaMascus P., Zheng L., Jackson A., Wiacek R. J., Carpick R. W. (2020). Cooperativity Between
Zirconium Dioxide Nanoparticles
and Extreme Pressure Additives in Forming Protective Tribofilms: Toward
Enabling Low Viscosity Lubricants. Tribol. Lett..

[26] Sharma V., Timmons R. B., Erdemir A., Aswath P. B. (2019). Interaction of Plasma
Functionalized TiO2 Nanoparticles and ZDDP on Friction and Wear under
Boundary Lubrication. Appl. Surf. Sci..

[27] Vyavhare K., Timmons R. B., Erdemir A., Edwards B. L., Aswath P. B. (2021). Robust
Interfacial Tribofilms by Borate- A Nd Polymer-Coated ZnO Nanoparticles
Leading to Improved Wear Protection under a Boundary Lubrication Regime. Langmuir.

[28] Delghandi D., Fulco S., Nautiyal P., LaMascus P., Wiacek R. J., Turner K. T., Carpick R. W. (2023). Metal Oxide
Tribofilms: Relating
Antiwear Additive Synergy with Mechanical Properties. Tribol. Lubr. Technol..

[29] Thrush S. J., Comfort A. S., Dusenbury J. S., Han X., Barber G. C., Wang X., Qu H. (2021). Wear Mechanisms of a Sintered Tribofilm
in Boundary Lubrication Regime. Wear.

[30] Groza J. R., Zavaliangos A. (2000). Sintering
Activation by External Electrical Field. Mater.
Sci. Eng., A.

[31] Li W., Wu D., Hu K., Xu Y., Yang X., Zhang Y. (2021). A Comparative
Study on the Employment of Heat Treatment, Electric Pulse Processing
and Friction Stir Processing to Enhance Mechanical Properties of Cold-Spray-Additive-Manufactured
Copper. Surf. Coat. Technol..

[32] Liu Z., Wang H., Wang Y., Tian L., Li H., Liu W., He P., Liu H., Li R. (2023). Comparative Study on
the Annealing of Cold-Sprayed Boron Nitride Nanosheet/Copper Coating
Using Spark Plasma Sintering and Atmosphere Furnace. Surf. Coat. Technol..

[33] Sun W., Bhowmik A., Tan A. W.-Y., Li R., Xue F., Marinescu I., Liu E. (2019). Improving Microstructural and Mechanical
Characteristics of Cold-Sprayed Inconel 718 Deposits via Local Induction
Heat Treatment. J. Alloys Compd..

[34] Shimizu Y., Spikes H. A. (2016). The Influence of
Slide–Roll Ratio on ZDDP Tribofilm
Formation. Tribol. Lett..

[35] Smeeth, M. ; Hamer, C. ; Spikes, H. A. A Study of Antiwear Additive Film Build Up Using the MTM (Mini-Traction Machine). In ASME/STLE 2007 International Joint Tribology Conference, Parts A and B; ASMEDC, 2007; pp 101–103.

[36] LaMascus, P. Tribosintering of Metal Oxide Nanocrystals; University of Pennsylvania, 2025. http://argo.library.okstate.edu/login?url=https://www.proquest.com/dissertations-theses/tribosintering-metal-oxide-nanocrystals/docview/3218347983/se-2?accountid=4117.

[37] Munir Z. A., Quach D. V., Ohyanagi M. (2011). Electric Current
Activation of Sintering:
A Review of the Pulsed Electric Current Sintering Process. J. Am. Ceram. Soc..

[38] Guillon O., Gonzalez-Julian J., Dargatz B., Kessel T., Schierning G., Räthel J., Herrmann M. (2014). Field-Assisted Sintering Technology/Spark
Plasma Sintering: Mechanisms, Materials, and Technology Developments. Adv. Eng. Mater..

[39] Dash A., Liu Y., Yoshida H., Mücke R., Gerstein G., Herbst S., Nürnberger F., Maier H. J., Han H. N., Lin S., Guillon O. (2025). Electroplasticity
of Metals and Ceramics: Current Status. Annu.
Rev. Mater. Res..

[40] Conrad H., Sprecher A. F., Cao W. D., Lu X. P. (1990). Electroplasticity—the
Effect of Electricity on the Mechanical Properties of Metals. JOM.

[41] Girão, A. V. ; Caputo, G. ; Ferro, M. C. Application of Scanning Electron Microscopy–Energy Dispersive X-Ray Spectroscopy (SEM-EDS). In Characterization and Analysis of Microplastics; Elsevier, 2017; pp 153–168.

[42] Stanciu V. I., Erauw J.-P., Boilet L., Vitry V., Delaunois F. (2023). WC-Co Composite
Made with Doped Binder: The Effect of Binder Proportion on Microstructure
and Mechanical Properties. Int. J. Refract.
Met. Hard Mater..

[43] Schwesig D., Schierning G., Theissmann R., Stein N., Petermann N., Wiggers H., Schmechel R., Wolf D. E. (2011). From Nanoparticles
to Nanocrystalline Bulk: Percolation Effects in Field Assisted Sintering
of Silicon Nanoparticles. Nanotechnology.

[44] La
Barbera-Sosa J. G., Santana Y. Y., Caro J., Chicot D., Lesage J., Staia M. H., Puchi-Cabrera E. S. (2014). Mechanical
Properties of WC Coatings Evaluated Using Instrumented Indentation
Technique. Surf. Eng..

[45] Li J., Lei G., Yu G., Cao X., Xia Y., Yan S., Xiao Q., Ye X.-X. (2025). Ni-Substituted
WC Cemented Carbides
with Chromium Carbide Coatings: Enhanced High-Temperature Oxidation
Resistance. Surf. Coat. Technol..

[46] Nam S., Song Y., Son M., Lee K.-A., Yoo G. H., Park E. S., Choi H., Lee C. S. (2021). High-Temperature
Mechanical and Thermochemical Properties of NbMoTaW Refractory High-Entropy
Alloy Coatings Produced via Nano Particle Deposition System (NPDS). Int. J. Refract. Met. Hard Mater..

[47] Li H., Cao F., Li T., Tan Y., Chen Y., Wang H., Liaw P. K., Dai L. (2024). Enhanced Plasticity
in Refractory
High-Entropy Alloy via Multicomponent Ceramic Nanoparticle. J. Mater. Sci. Technol..

[48] Liu Y., Zhang Y., Zhang H., Wang N., Chen X., Zhang H., Li Y. (2017). Microstructure and Mechanical Properties
of Refractory HfMo0.5NbTiV0.5Six High-Entropy Composites. J. Alloys Compd..

