# Supplementary material for: Low-Temperature Fabrication of Refractory Thin Films via Electric Field and Contact Stress-Activated Sintering of Nanoparticles: An In Situ Study
Source: Nano Lett. 2025 Nov 17;25(47):16740–6. doi: 10.1021/acs.nanolett.5c04675 (PMC12670486; doi:10.1021/acs.nanolett.5c04675)
Supplement: Supplementary file 1 [file nl5c04675_si_001.pdf]

Supporting Information

Low-Temperature Fabrication of Refractory Thin Films via Electric Field and Contact Stress-Activated Sintering of Nanoparticles: An *In Situ* Study

Bunty Tomar, Pranjal Nautiyal*

School of Mechanical and Aerospace Engineering, Oklahoma State University, Stillwater, OK 74078, USA.

*Corresponding Author: pranjal.nautiyal@okstate.edu

Content:

S1. Methods

S2. Materials

S3. Figures S1 to S8

S4. References

S1. Methods

S1.1. In-situ film growth experiments

In this study, we used a ball-on-disc tribometer (Mini Traction Machine, PCS Instruments, London, UK), capable of generating high-pressure contact conditions, to investigate sintering of WC-Co nanoparticles. This machine can simulate a wide range of slide-roll ratios, from pure rolling to mixed sliding/rolling to pure sliding[1]. The tribometer was equipped with a 3-D space layer imaging (SLIM)[2] system for in-situ measurement of film thickness during tribosintering experiments. For these measurements, the test is intermittently stopped, the ball is lifted off the disc, and pressed against a glass window with a semi-reflective chromium layer to capture the optical interference images at regular intervals[3]. The interference image is then correlated to film thickness using SLIM offline analyses software, which relies on a calibration chart of RGB color versus optical path difference[4]. A refractive index of 3 was assumed for the WC-Co coating[5]. The SLIM system was calibrated to capture optical image of the same defined location at every imaging cycle. The ball-on-disc mini traction machine was modified to apply a direct current (DC) across the ball/disc interface during film formation. The ball and disc shafts shown in **figure 1**, were electrically isolated from the rest of the rig using custom-designed insulating sleeves. This ensured that the current could flow exclusively through the ball and disc contact only[6]. The DC power source was synchronized with the contact cycles and turned on only during contact cycles, i.e. during ball and disc engagement and was turned off during ball lift off and interference imaging. The test conditions selected are shared in **Table S1**.

Table S1. Ball-on-disc tribometry test conditions

**Parameters**	**Condition**
Load	50 N
Maximum Hertzian contact pressure	1.12 GPa
Mean/ Entrainment speed	100 mm/s
Slide-to-roll ratio (*SRR*)	5%
Carrier fluid temperature	100°C
Electric current	0, 2, 5 A

The ratio of the sliding speed to the mean rolling speed is known as slide roll ratio (SRR),

$$SRR = \frac{|u_D - u_B|}{U} \qquad \text{(S1)}$$

where, u_B and u_D are the speed of ball and disc relative to the surface, respectively. U is the mean speed: $\frac{u_B+u_D}{2}$.

Hertz contact mechanics model[7] was used to estimate the contact pressure between ball and disc. In Hertz model, the maximum contact pressure (σ) is given by:

$$\sigma = \frac{3F}{2\pi a^2} \qquad \text{(S2)}$$

where, F is the given normal load and a is contact radius given by:

$$a = \left(\left(\frac{3FR}{4E^*}\right)^{\frac{1}{3}}\right) \qquad \text{(S3)}$$

where R is the radius of the ball.

E^* is the reduced Young's modulus:

$$\frac{1}{E^*} = \frac{(1-v_1^2)}{E_1} + \frac{(1-v_2^2)}{E_2} \qquad \text{(S4)}$$

where, E_1, E_2 are Young's modulus and v_1, v_2 are Poisson's ratio of ball and disc, respectively.

The film thickness vs. time plots for the initial growth phase fitted well to a power-law function,

$$h(t) = at^b \qquad \text{(S5)}$$

where, h is the average film thickness in nm; t is time in seconds, and a, b are the fitting parameters. The film thickness evolution plots in **Figure 2a** were fitted using the OriginPro 2024 software to obtain the fitting parameters, a and b.

To determine the film growth rates, the power law fit was differentiated with respect to time[8],

$$\frac{dh}{dt} = abt^{(b-1)} \qquad \text{(S6)}$$

Using this equation, instantaneous film growth rates were calculated for each data point in the growth regime (shown in Figure 2a), and the mean growth rate was then determined by averaging these values. **Figure 2c** presents the calculated average film growth rates as a function of the applied direct current.

S1.2. Thin film characterization

All the thin film samples were sonicated and washed in toluene followed by isopropanol before carrying out characterization. Atomic Force Microscopy, a high-resolution surface characterization technique was used to image the topography of the films on the disc specimen using an atomic force microscope (AFM; Asylum Research MFP3D Infintiy, Santa Barbara, CA, USA). A sharp silicon tip with radius of curvature less than 5 nm was used to image the thin film surface and Gwyddion (version 2.67) software was used for image processing and data analysis.

The microstructure and chemical composition of the coating were characterized by a field emission scanning electron microscope (FESEM; FEI Quanta 600, OR, USA) and energy dispersive X-ray spectroscopy (EDS; QUANTAX, Bruker, Billerica, MA, USA). A beam energy of 20 kV and a working distance of 10 mm were used for FESEM analyses.

The cross-section of the film, as well as the bonding between the film and the substrate, were characterized using focused ion beam milling-scanning electron microscopy (FIB-SEM; Thermo Scientific Scios 2 DualBeam, MA, USA). The sample was milled with a Ga^{+} ion beam operating at a beam current of 3.2 nA and an accelerating voltage of 30 kV. The sample stage was tilted to an angle of 52° for milling and subsequent SEM imaging. A trench measuring approximately 4 μm in length, 2 μm in width, and 2 μm in depth was created. SEM imaging and EDS analyses of the exposed cross-section were performed with a beam operating at an accelerating voltage of 10 kV and with a working distance of 7 mm.

S2. Materials

The WC-Co (5wt.% Co) nanoparticles of size 100-200 nm and WC nanoparticles of size 20-120 nm (US Research Nanomaterials, Houston, TX, USA) were used in this study. Nanoparticles were dispersed in a low viscosity synthetic polyalphaolefin (PAO2) oil (Exxon Mobil, USA) using a surfactant, which reduces the particle-particle adhesion, subsequently preventing nanoparticle agglomeration. In this study, polyisobutylene succinimide (PIBS) (Dorf Ketal, USA), was used as a surfactant to disperse WC-Co nanoparticles[9] in the PAO2 oil. The solution was magnetically stirred for 45 minutes, followed by 8 hours of tip sonication (Sonics VCX 750) with the probe (6 mm tapered microtip) frequency of 20 kHz at 40 μm amplitude.

The PAO oil serves as a carrier fluid, and does not participate in film formation. It is stable and does not react or evaporate at the 100°C temperature used in this work.

The ball and disc material for tribosintering experiments was 52100 bearing steel, with a root mean square (RMS) surface roughness of ~5 nm.

S3. Figures

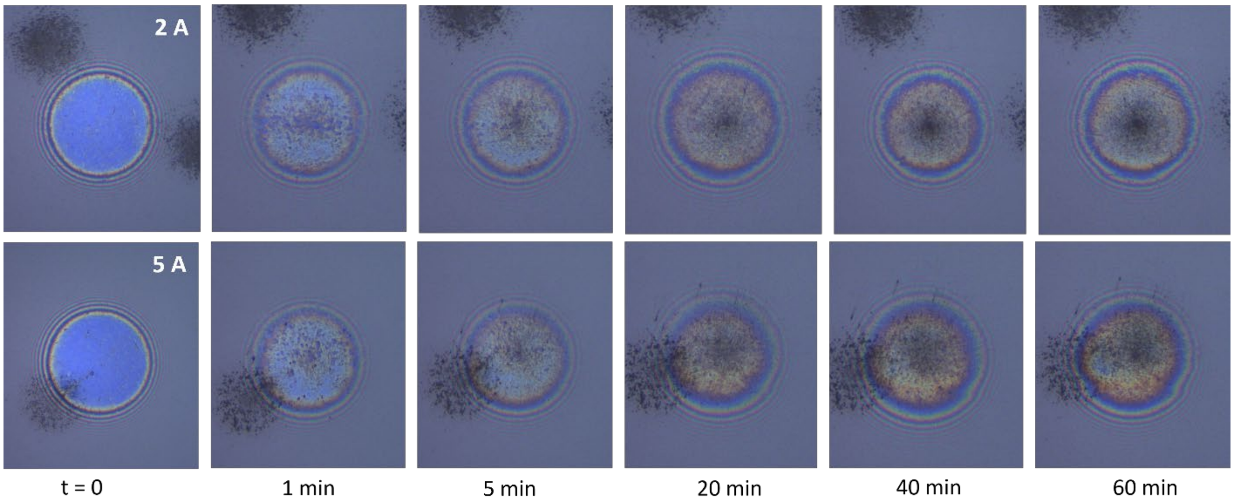

Fig. S1 | SLIM images showing the growth of WC-Co films. In-situ optical interferometry images showing nucleation and growth of WC-Co film on the ball specimen under 2 and 5 A conditions.

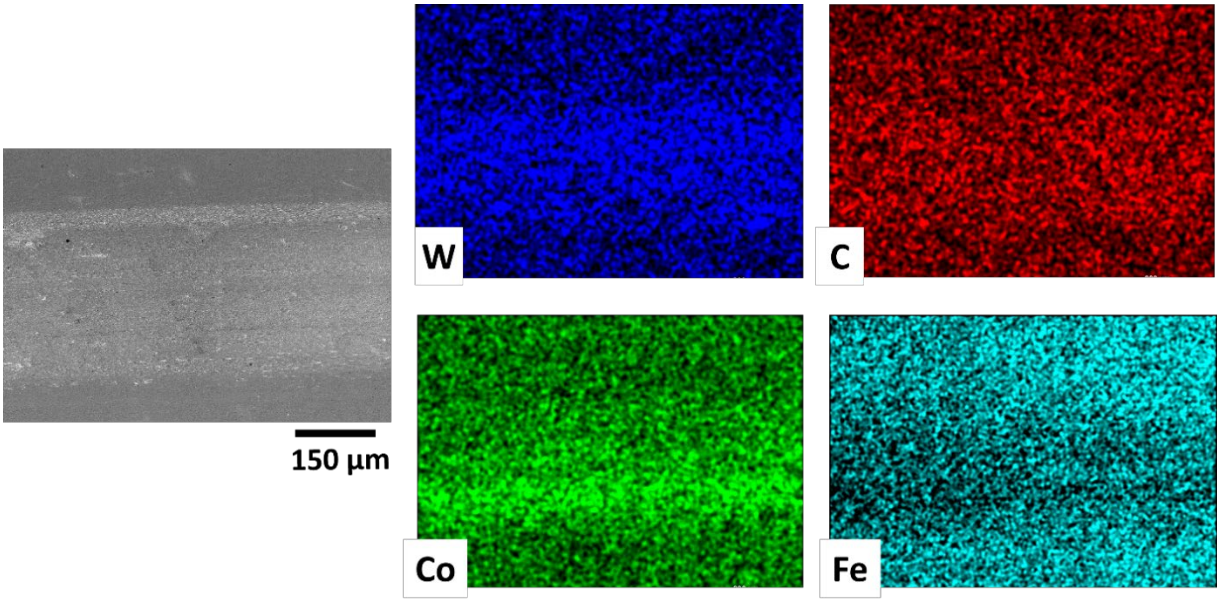

Fig. S2 | EDS maps of WC-Co thin films manufactured at 5 A electric field condition. The presence of W, C, and Co signals confirm successful formation of WC-Co thin films.

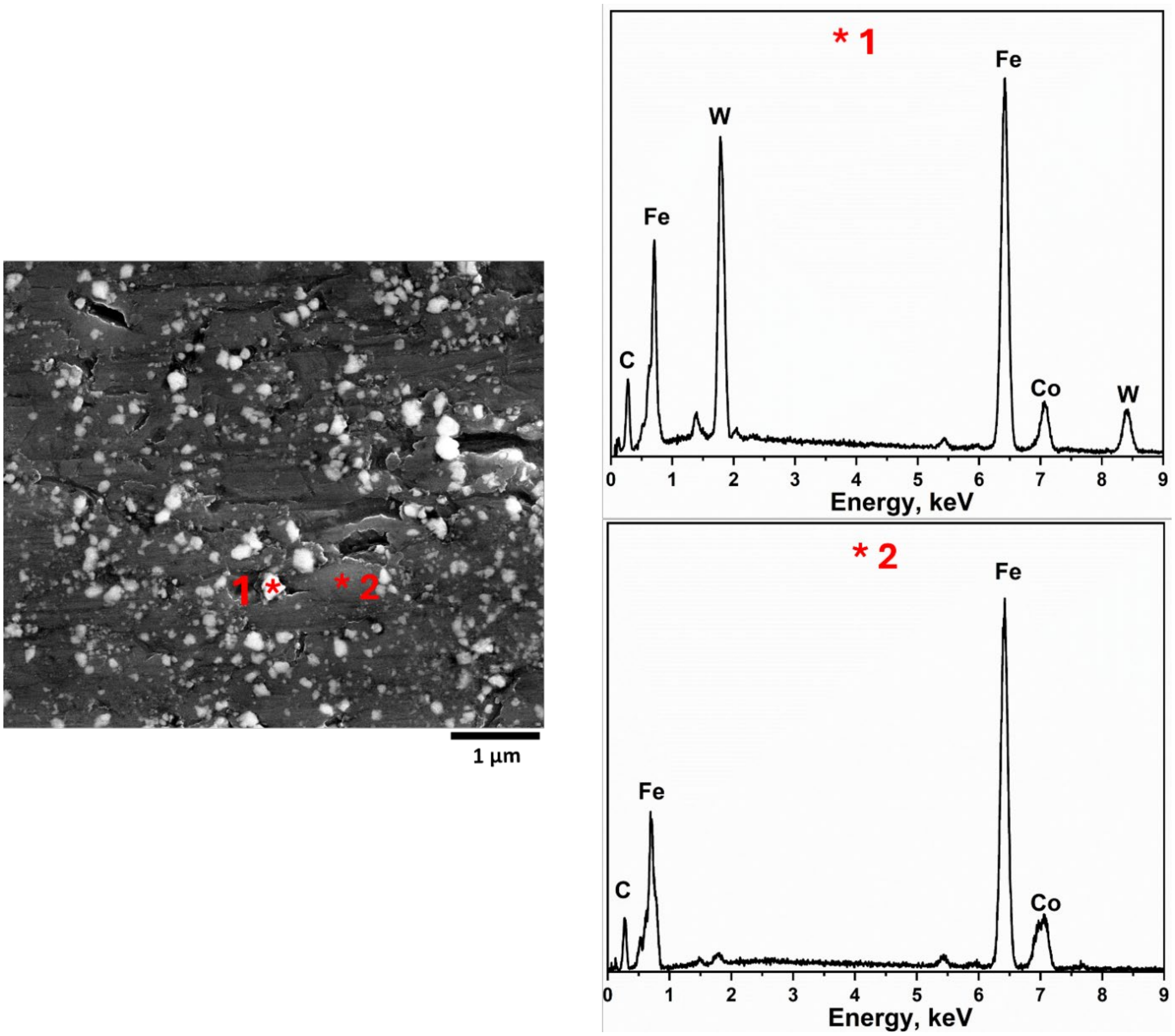

Fig. S3 | Point EDS results of WC-Co thin films manufactured at 5 A electric field conditions. Elemental spectra from point 1 (white particle) reveal strong W peaks, confirming the presence of WC.

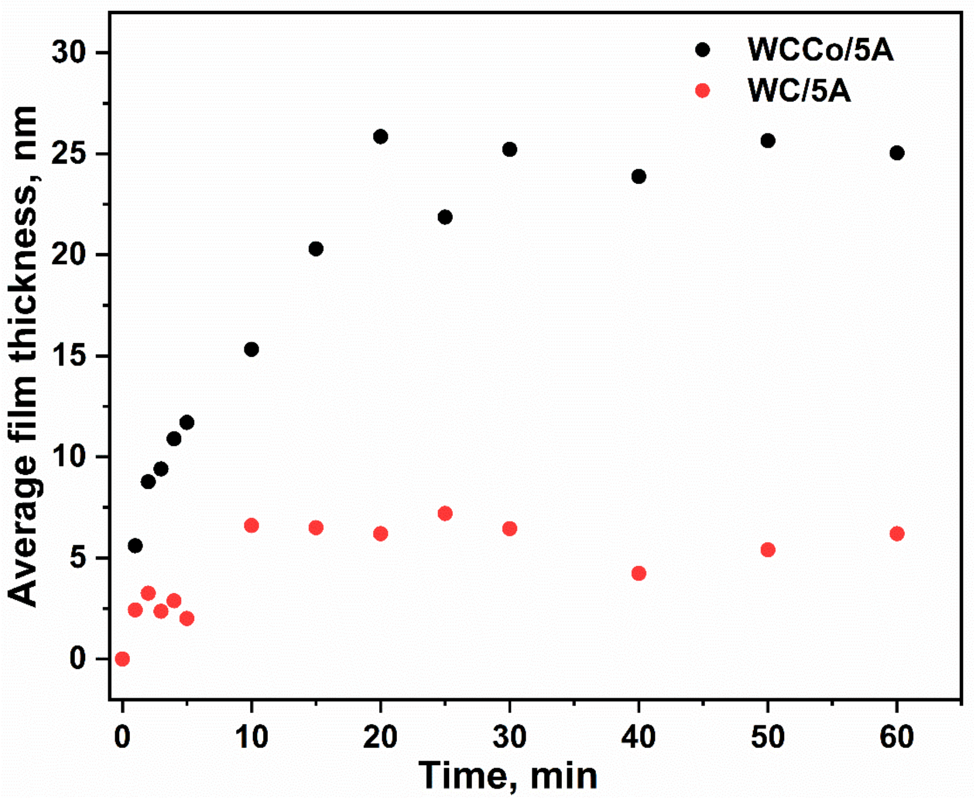

Fig. S4 | Comparison of WC-Co vs WC average film thickness at 5 A test conditions. The WC nanoparticles show significantly reduced film thickness (~5 nm) compared to WC-Co films (~25 nm), highlighting the critical role of the ductile Co phase in facilitating electric field assisted tribosintering.

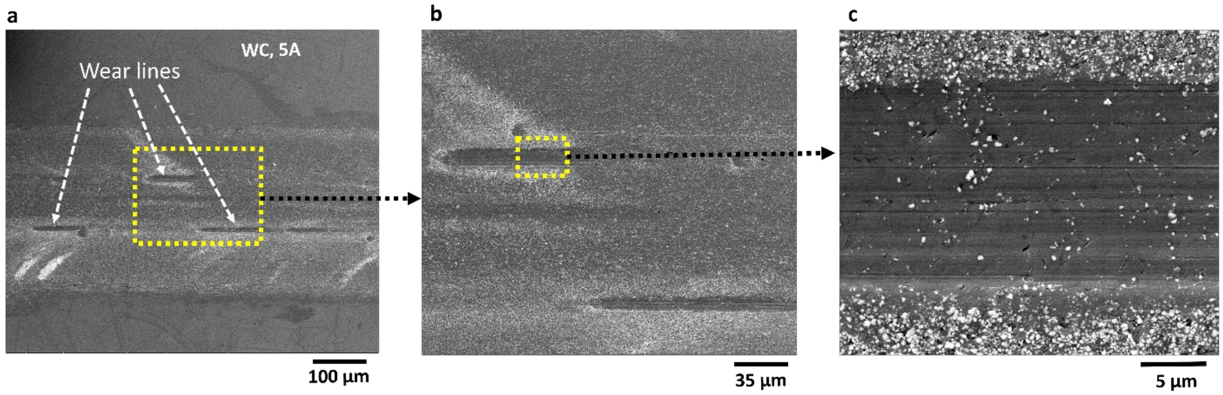

Fig. S5 | SEM images of WC films manufactured under 5 A electric field conditions. The film surface exhibits extensive wear scars exceeding 100 µm in length and ~15 µm in width, indicating severe abrasive interaction between WC nanoparticles and the substrate.

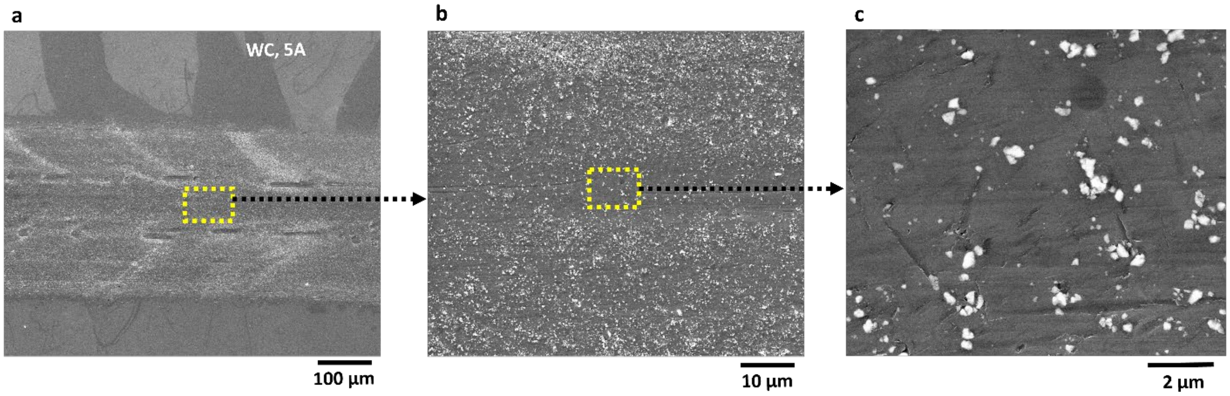

Fig. S6 | Microstructural features of WC-only films formed under 5 A electric current conditions. a, prominent wear scars and limited surface coverage indicate poor film formation, likely due to inadequate nanoparticle consolidation. **b,c,** highlight the sparse and uneven distribution of WC nanoparticles across the surface.

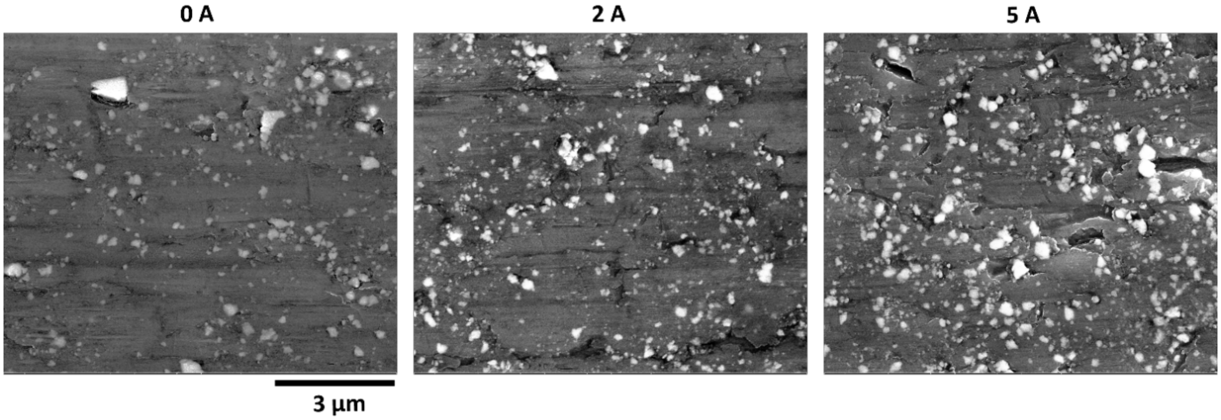

Fig. S7 | SEM images of WC-Co thin films manufactured under 0 A, 2 A and 5 A test conditions. The progressive increase in distribution of white WC particles on the top surface of thin films is evident with increasing current, highlighting electric field-induced compositional evolution.

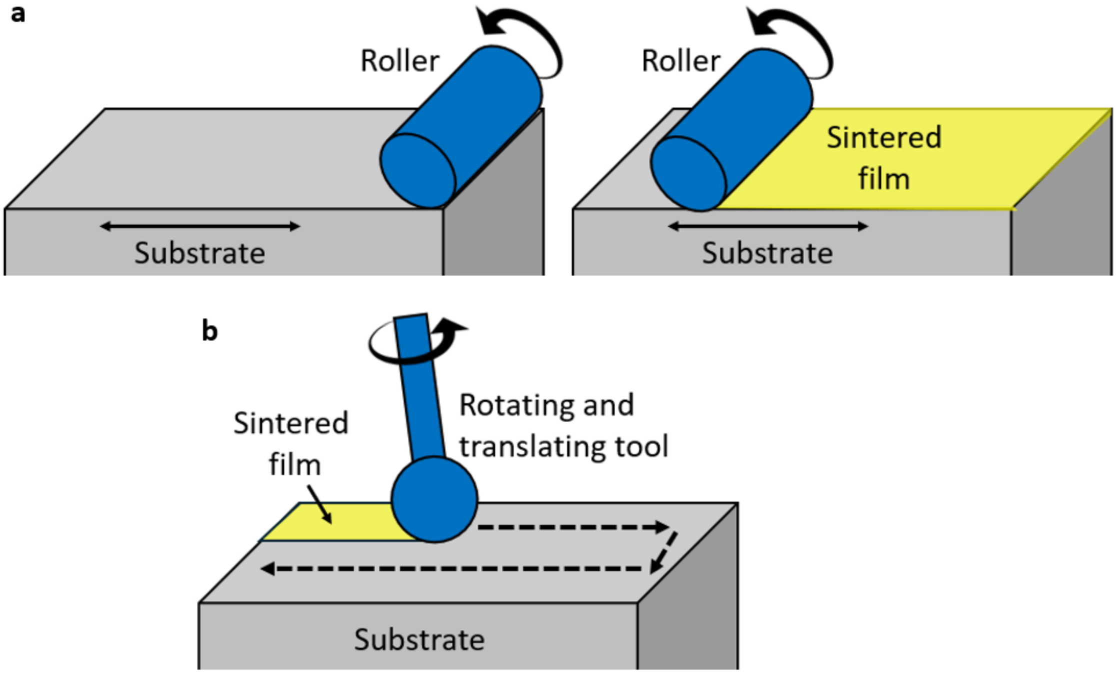

Fig. S8 | Schematic representation of strategies for scaling this method to coat large areas. a, Cylinder-on-flat contact which generates a line contact with substantially greater contact area for field-assisted tribosintering. **b,** A rotating probe can traverse over the substrate (as indicated by the dashed lines) to sinter films over large areas.

S4. References

(1) Shimizu, Y.; Spikes, H. A. The Influence of Slide–Roll Ratio on ZDDP Tribofilm Formation. *Tribol. Lett.* **2016**, *64* (2), 19. https://doi.org/10.1007/s11249-016-0738-z.

(2) Cann, P. M.; Spikes, H. A.; Hutchinson, J. The Development of a Spacer Layer Imaging Method (SLIM) for Mapping Elastohydrodynamic Contacts. *Tribol. Trans.* **1996**, *39* (4), 915–921. https://doi.org/10.1080/10402009608983612.

(3) Smeeth, M.; Hamer, C.; Spikes, H. A. A Study of Antiwear Additive Film Build Up Using the MTM (Mini-Traction Machine). In *ASME/STLE 2007 International Joint Tribology Conference, Parts A and B*; ASMEDC, 2007; pp 101–103. https://doi.org/10.1115/IJTC2007-44249.

(4) Dawczyk, J.; Morgan, N.; Russo, J.; Spikes, H. Film Thickness and Friction of ZDDP Tribofilms. *Tribol. Lett.* **2019**, *67* (2), 34. https://doi.org/10.1007/s11249-019-1148-9.

(5) Romanus, H.; Cimalla, V.; Schaefer, J. .; Spieß, L.; Ecke, G.; Pezoldt, J. Preparation of Single Phase Tungsten Carbide by Annealing of Sputtered Tungsten-Carbon Layers. *Thin Solid Films* **2000**, *359* (2), 146–149. https://doi.org/10.1016/S0040-6090(99)00732-4.

(6) Yousuf, A.; Spikes, H.; Guo, L.; Kadiric, A. Influence of Electric Potentials on Surface Damage in Rolling–Sliding Contacts Under Mixed Lubrication. *Tribol. Lett.* **2025**, *73* (2), 45. https://doi.org/10.1007/s11249-025-01977-2.

(7) Johnson, K. L. *Contact Mechanics*; Cambridge University Press, 1985. https://doi.org/10.1017/CBO9781139171731.

(8) Ernens, D.; Langedijk, G.; Rooij, P. S. M. B. De; Schipper, H. R. P. D. J. Characterization of the Adsorption Mechanism of Manganese Phosphate Conversion Coating Derived Tribofilms. *Tribol. Lett.* **2018**, *66* (4), 1–16. https://doi.org/10.1007/s11249-018-1082-2.

(9) Armstrong, R. W. The Hardness and Strength Properties of WC-Co Composites. *Materials (Basel).* **2011**, *4* (7), 1287–1308. https://doi.org/10.3390/ma4071287.
